# Surgery for Aortic Prosthetic Valve Endocarditis in the Transcatheter Era

**DOI:** 10.3390/jcm11123418

**Published:** 2022-06-14

**Authors:** Shekhar Saha, Ahmad Ali, Philipp Schnackenburg, Konstanze Maria Horke, Andreas Oberbach, Nadine Schlichting, Sebastian Sadoni, Konstantinos Rizas, Daniel Braun, Maximilian Luehr, Erik Bagaev, Christian Hagl, Dominik Joskowiak

**Affiliations:** 1 Department of Cardiac Surgery, Ludwig Maximillian University of Munich, Marchioninistrasse 15, 81377 Munich, Germany; ahmad.ali@med.uni-muenchen.de (A.A.); philipp.schnackenburg@med.uni-muenchen.de (P.S.); konstanze.horke@med.uni-muenchen.de (K.M.H.); andreas.oberbach@medizin.uni-leipzig.de (A.O.); sebastian.sadoni@med.uni-muenchen.de (S.S.); maximilian.luehr@uk-koeln.de (M.L.); erik.bagaev@klinikum-nuernberg.de (E.B.); christian.hagl@med.uni-muenchen.de (C.H.); dominik.joskowiak@med.uni-muenchen.de (D.J.); 2 German Centre for Cardiovascular Research (DZHK), Partner Site Munich Heart Alliance, 80802 Munich, Germany; 3 Department of Diagnostics, Fraunhofer Institute for Cell Therapy and Immunology, 04109 Leipzig, Germany; nadine.schlichting@medizin.uni-leipzig.de; 4 Institute of Clinical Immunology, University of Leipzig, 04109 Leipzig, Germany; 5 German Centre for Cardiovascular Research (DZHK), Department of Cardiology, Ludwig Maximillian University of Munich, Partner Site Munich Heart Alliance, 80802 Munich, Germany; konstantinos.rizas@med.uni-muenchen.de (K.R.); daniel.braun@med.uni-muenchen.de (D.B.); 6 Department of Cardiothoracic Surgery, University Hospital Cologne, Kerpener Str. 62, 50937 Cologne, Germany; 7 Department of Cardiac Surgery, Paracelsus Medical University, 90419 Nuremberg, Germany

**Keywords:** infective endocarditis, transcatheter aortic valve implantation, prosthetic valve endocarditis

## Abstract

*Objectives*: As surgical experience with infective endocarditis following transcatheter aortic valve replacement is scarce, this study compared the perioperative and short-term outcomes of patients suffering from endocarditis following surgical aortic valve replacement and transcatheter aortic valve replacement. *Methods*: Between January 2013 and December 2020, 468 consecutive patients were admitted to our center for surgery for IE. Among them, 98 were operated on for endocarditis following surgical aortic valve replacement and 22 for endocarditis following transcatheter aortic valve replacement. *Results*: The median EuroSCORE II (52.1 (40.6–62.0) v/s 45.4 (32.6–58.1), *p* = 0.207) and STS-PROM (1.8 (1.6–2.1) v/s 1.9 (1.4–2.2), *p* = 0.622) were comparable. Endocarditis following transcatheter aortic valve replacement accounted for 13.7% of the aortic prosthetic valve endocarditis between 2013 and 2015; this increased to 26.9% in the years 2019 and 2020.Concomitant procedures were performed in 35 patients (29.2%). The operative mortality was 26.5% in the endocarditis following surgical aortic valve replacement group and 9.1% in the endocarditis following transcatheter aortic valve replacement group (*p* = 0.098). Upon follow-up, survival at 6 months was found to be 98% in the group with endocarditis following surgical aortic valve replacement and 89% in the group with endocarditis following transcatheter aortic valve replacement (*p* = 0.081). *Conclusions*: Patients suffering from endocarditis following surgical aortic valve replacement and transcatheter aortic valve replacement present with comparable risk profiles and can be surgically treated with comparable results. Surgery as a curative option should not be rejected even in this intermediate-risk cohort.

## 1. Introduction

Although current guidelines consider surgery to be the best option in cases of prosthetic valve endocarditis (PVE), the current literature reports a general reluctance toward the surgical treatment of IE following transcatheter aortic valve implantation (TIE), with some patients entering palliative care upon diagnosis [1,2,3,4,5]. Infective endocarditis (IE) is the leading cause of reoperation following transcatheter aortic valve replacement [6]. Up to 90% of patients suffering from TIE undergo conservative treatment, and this has been associated with high in-hospital mortality and poor short-term survival [7]. Along with clinical evaluation, risk scores play an important role in the decision-making process. Endocarditis-specific risk scores have been reported to have better prognostic performance than classical risk scores, as they take into consideration specific factors such as microbiological cultures, abscess formation, and sepsis [8]. Although surgery for aortic PVE entails a high rate of early morbidity and mortality, survivors exhibit satisfactory long-term survival, with a low risk of recurrent endocarditis [9]. With the rise in the number of transcatheter aortic valve replacements (TAVRs) and the indubitable rise in TIE, an indisposition to surgical therapy can be disastrous. As surgical experience with TIE is scarce, this study compares the perioperative and short-term outcomes of patients suffering from PVE following surgical aortic valve replacement (SAVR–PVE) and TIE.

## 2. Patients and Methods

### 2.1. Study Design

We reviewed the IE case load at our center between January 2013 and December 2020 and retrospectively identified patients who underwent surgery for IE following SAVR and TAVR. Postoperative treatment and data acquisition were performed as part of routine patient care. This study was approved by the ethics board of the Ludwig Maximilian University (Nos. 19-730 and 20-821), and the requirement to obtain patient consent was waived for this retrospective study. Sampling and microbiological analysis are described elsewhere [10]. Data acquisition was based on institutional databases, and the data were then de-identified. We analyzed the patient characteristics, individual risk scores, surgical details, and postoperative and early outcomes of these patients.

### 2.2. Preoperative Risk Determination

To predict the postoperative mortality, the European System for Cardiac Operative Risk Evaluation II (EuroSCORE II), as proposed by Nashef et al. [11], and the Society of Thoracic Surgeons Predicted Risk of Mortality (STS PROM) score were calculated. Furthermore, endocarditis-specific scores such as the Endoscore, as proposed by Di Mauro et al. [12]; the Risk E score, as proposed by Olmos et al. [13]; and the De Feo Score [14] were calculated. The ICE Score, as proposed by Park et al. [15], was used to predict the 6-month mortality following surgery for IE.

### 2.3. Data Collection, Statistical Analysis, and Illustrations

Data were analyzed by using IBM SPSS version 25 (Statistical Package for the Social Sciences) (IBM-SPSS Inc., Armonk, NY, USA). Categorical variables were evaluated by using the Chi-Squared and Fisher’s exact method, and continuous variables were evaluated by using the Mann–Whitney U test. Survival analysis was performed with a Kaplan–Meier curve and log-rank test. Data are presented as medians (25th–75th quartiles) or absolute values (percentages), unless otherwise specified. Illustrations were prepared by using GraphPad Prism (GraphPad Software Inc., San Diego, CA, USA) and BioRender (BioRender, San Diego, CA, USA).

## 3. Results

### 3.1. Patient Population

**Figure 1:**
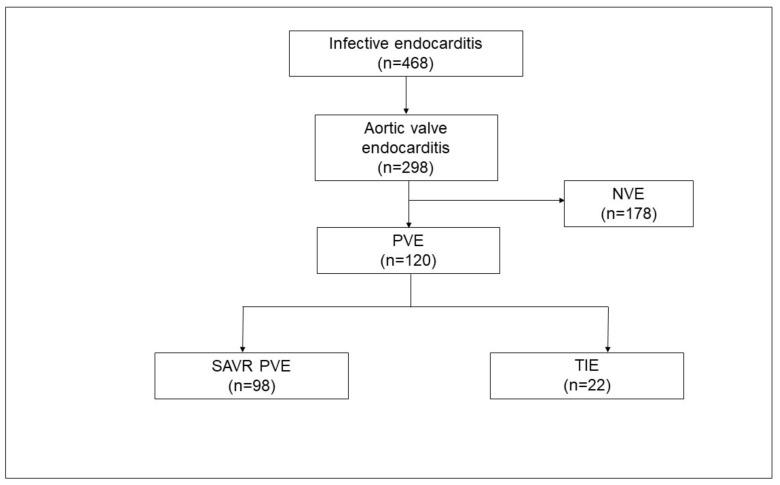
Case load of infective endocarditis from January 2013 to December 2020. NVE, native valve endocarditis; PVE, prosthetic valve endocarditis; SAVR–PVE, prosthetic valve endocarditis following surgical aortic valve replacement; TIE, prosthetic valve endocarditis following transcatheter aortic valve replacement.

Between January 2013 and December 2020, 468 consecutive patients were admitted to our center for surgery for IE (Figure 1). Among these patients, 120 patients were operated on for aortic prosthetic valve endocarditis. IE following SAVR (SAVR–PVE) was diagnosed in 98 patients, and IE following TAVR (TIE) was diagnosed in 22 patients.

**Figure 2:**
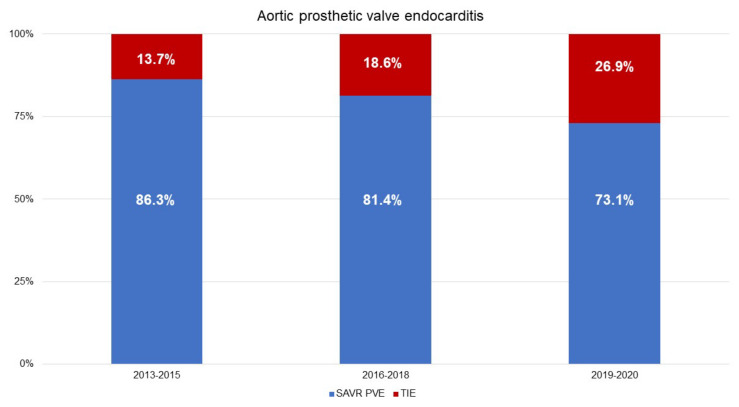
Temporal distribution of aortic prosthetic valve endocarditis with the distribution of SAVR–PVE and TIE. (Data are expressed as percentages.) SAVR–PVE, prosthetic valve endocarditis following surgical aortic valve replacement; TIE, prosthetic valve endocarditis following transcatheter aortic valve replacement.

TIE accounted for 13.7% of the aortic PVE between 2013 and 2015; this increased to 26.9% in the years 2019 and 2020 (Figure 2). The demographic characteristics are presented in Table 1. The median age was 69 years (58–76 years) in the SAVR–PVE group and 77 years (70–80 years) in the TIE group (*p* = 0.010). The median Charlson Comorbidity Index was 5 (4–7) in the SAVR–PVE group and 7 (6–8) in the TIE group (*p* = 0.005).

In the TIE group, nine patients (40.9%) had undergone previous cardiac surgery (*p* < 0.001). In the SAVR–PVE group, 84 (85.7%) patients had biological prostheses, 13 (13.3%) had mechanical prostheses, and 1 (1.0%) patient had a homograft. A stentless pericardial prosthesis was implanted in one patient. In the TAVR group, 3 (13.6%) patients had received self-expanding prostheses (CoreValve: *n* = 3), and 19 patients (86.4%) had received balloon expanding prostheses (Sapien XT: *n* = 5, Sapien 3: *n* = 14). A significantly higher proportion of patients in the TIE group had pacemakers (9 (40.9%) v/s 12 (12.2%); *p* = 0.003) and were on chronic steroid therapy (3 (13.6%) v/s 1 (1.0%), *p*= 0.019) as compared to the SAVR–PVE group. The time to IE was significantly longer in patients who underwent SAVR as compared to those who underwent TAVR (3.7 years (0.8–9.5 years) v/s 1.2 years (0.4–2.8 years); *p* = 0.001). The classical risk scores and the endocarditis-specific risk scores, as described above, are detailed in Table 2. No differences were observed between the groups. Preoperative echocardiographic data are depicted in Table 3. Paravalvular leakage was observed in 12 patients (12.2%) in the SAVR–PVE group and in 8 patients (36.4%) in the TIE group (*p* = 0.013).

### 3.2. Causative Organisms

BCNIE accounted for 27 cases (27.8%) in the SAVR–PVE group and 1 case (4.5%) in the TIE group (*p* = 0.024). The causative organisms in our cohort were predominantly Gram-positive, with Gram-negative organisms accounting for 2.5% of the cases. The details of the individual pathogens are outlined in Table 4.

### 3.3. Surgical Data

The details of surgery are presented in Table 5. In all cases, the TAVR prostheses were explanted. The median cardiopulmonary bypass time was 203 min (149–271 min) in the SAVR–PVE group and 127 min (87–232 min) in the TIE group (*p* = 0.005). The duration of aortic cross-clamping was 134 min (106–169 min) in the SAVR–PVE group and 95 min (58–168 min) in the TIE group (*p* = 0.003). Biological prostheses were used in 91 patients (92.9%) in the SAVR–PVE group and in 20 patients (90.9%) in the TIE group, whereas mechanical prostheses were used in 7 patients (7.1%) in the SAVR–PVE group and in 2 patients (9.1%) in the TIE group. Bentall procedures were carried out in 34 patients (34.7%) in the SAVR–PVE group and in 1 patient (4.5%) in the TIE-group (*p* = 0.004). Stentless xenopericardial prostheses were used in 30 patients (30.6%) in the SAVR–PVE group and in 1 patient (4.5%) in the TIE group. One patient in the SAVR–PVE group received a Homograft. Repair of aortomitral curtain was required in two patients (2.0%) in the SAVR–PVE group, whereas it was required in only one patient (4.5%) in the TIE group (*p* = 0.458). A total of 19 patients (19.4%) required patch repair in the SAVR–PVE group, whereas 3 patients (13.6%) underwent the same in the TIE group (*p* = 0.762). Concomitant procedures were performed in 35 patients (29.2%) and are detailed in Table 5. No differences were observed between the groups.

### 3.4. Morbidities and Outcomes

**Figure 3:**
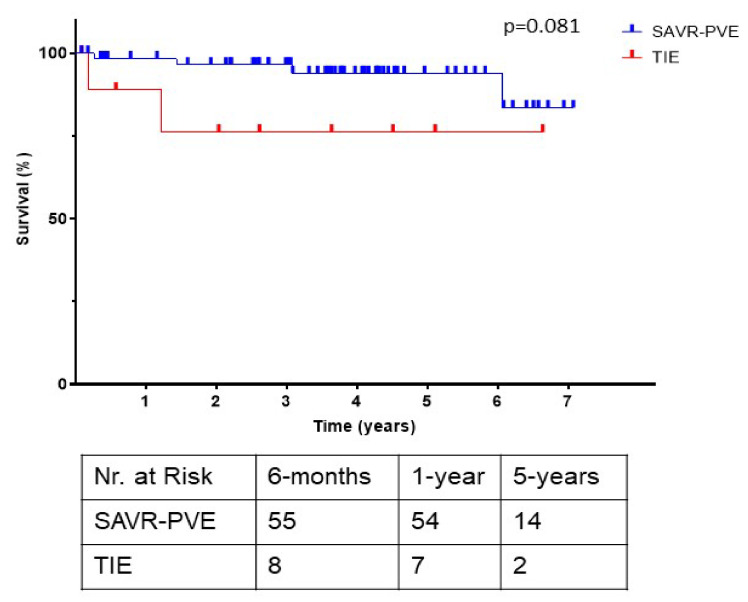
Kaplan–Meier survival curves of the SAVR–PVE group compared with the TIE group, with the number of patients at risk in tabular form. SAVR–PVE, prosthetic valve endocarditis following surgical aortic valve replacement; TIE, prosthetic valve endocarditis following transcatheter aortic valve replacement.

The postoperative morbidities and outcomes are listed in Table 5. There were no differences in the rates of adverse cerebrovascular events, tracheostomy, pacemaker implantation, renal replacement therapy, ECLS, and IABP support. The median hospital stay (19 days (14–33) v/s 23 days (16–37); *p* = 0.234) and median intensive care unit stay (5 days (2–9 days) v/s 4 days (3–14 days); *p* = 0.953) were comparable between the groups. The in-hospital mortality was 26.5% (*n* = 26) in the SAVR–PVE group and 9.1% (*n* = 2) in the TIE group (*p* = 0.098). Following discharge, the survival at 30 days was 100% in both groups, whereas the survival at 6 months was found to be 98% in the SAVR–PVE group and 89% in the TIE group (*p* = 0.081) (Figure 3).

## 4. Comment

The devastating nature of TIE has long since been recognized, not just due to the myriad of co-morbidities, but also due to surgical challenge associated with explantation of TAVR prostheses and lack of surgical experience [16,17]. The study at hand demonstrates that surgery can be a curative therapy in the setting of TIE, with results comparable to surgery for SAVR–PVE.

### 4.1. Surgical Considerations

The number of PVE cases in general and TIE cases in particular has been on the rise [10,18]. Between 2019 and 2020, TIE accounted for more than one-fourth of the cases of aortic PVE at our center (Figure 1). At the same time, the current literature reports that surgical explantation of the infected TAVR-prosthesis was performed in only 2–14% of TIE cases, despite clear indications for surgical intervention in more than 80% of patients [7]. The reasons not to operate are very diverse and generally include a high clinical and surgical risk, as well a limited life expectancy. IE is known to be associated with an elevated risk of in-hospital mortality, which may be as high as 40% in cases of PVE [1]. Malvindi et al. [7] report that the overall postoperative in-hospital mortality for patients undergoing surgery for TIE is 28%, whereas other studies report mortality rates as high as 50% [4,19]. In our cohort, the operative mortality in the TIE group was under 10%, and the rates of in-hospital mortality and early survival were comparable to patients suffering from SAVR–PVE. The endothelialization of the TAVR prosthesis by contacting aortic tissue, as well as calcifications and thrombus formation at the aortic root, makes explantation challenging [17]. Furthermore, explantation of the TAVR prosthesis in the setting of TIE might be further complicated due to the presence of abscesses and fragility of the tissue, especially in patients on chronic steroid therapy. Periannular abscesses have been reported in up to 12–25% of patients with TIE, and there is a similar rate of periannular complications in cases of SAVR–PVE, with 50–60% of patients presenting with an annular abscesses, fistulae, or false aneurysms [7]. In our cohort, abscesses were detected in more than a third of the patients. A significantly higher proportion of patients in the SAVR–PVE group underwent Bentall procedures, and this is reflected in the significantly longer duration of cardiopulmonary bypass and aortic cross-clamping. The destruction of the aortic root and abscess formation in these patients reflect, on the one hand, the severity of the underlying disease and, on the other, a delay in the diagnosis and referral for surgical treatment following the initial onset of symptoms. Timely diagnosis may allow for preservation of the aortic root and easier explantation of heart valve prostheses. The type of TAVR prostheses may also play a role in the development of TIE: the bulkier stent frame of self-expanding valves could serve as a nidus during bacteremia and may also irritate and damage the endothelium, making them more susceptible to TIE [3,20,21].

However, previous studies have shown that TAVR prostheses can be explanted with reasonable results and no damage to adjacent structures [6,17]. The superior outcomes in our study may be related to the management of our patients in a high-volume heart valve center with a multidisciplinary endocarditis team approach [22]. Early surgical treatment of complicated endocarditis improves outcomes when compared to medical therapy alone, and it can result in the reduction of 6-month mortality from 33 to 16% [23]. Furthermore, Perrotta et al. [9] report that aortic PVE is associated with a high rate of early complications and early mortality; however, patients who survive the immediate postoperative period demonstrate satisfactory long-term survival. This was reflected in our data, where the operative mortality was quite high; however, we found the long-term survival to be good (98% and 89%, respectively) (Figure 3).

### 4.2. Decision-Making and the Role of an Endocarditis Team

The endocarditis-team approach has been reported to result in early diagnosis, better management strategies and compliance in antimicrobial therapy, and fewer cases of renal failure and deaths by embolic events and multiple organ failure [22,24]. At our center, all cases of complicated IE are discussed in the endocarditis team, and a patient-centered approach is applied. The estimation of surgical risk is crucial to the surgical decision-making process. The use of classical risk scores in the setting of IE have been confirmed to have a suboptimal prognostic ability [8]. Endocarditis-specific scores could possibly predict mortality with higher accuracy than classic risk scores, as they include IE-specific factors that could impact mortality, and which are not addressed by the classical risk scores. The current guidelines acknowledge that no single operative risk score is perfect and place emphasis on the preoperative assessment of operative risk [1].

In our cohort, we observed a few differences in the preoperative-risk profile of the patients that may influence the decision-making process and partly explain the reluctance toward surgery [5,7]. Age is a crucial factor which plays a role in the decision-making process. In our cohort, patients suffering from TIE were significantly older than those suffering from SAVR–PVE, and this is not surprising. Although advanced age is associated with higher risk of death, longer hospital stay, and neurologic complications, it is dependent on the patients’ comorbidities, level of dependence, and nutritional and cognitive status; therefore, age itself should not be a contraindication to complex valve surgery [24,25]. Furthermore, we found that a significantly higher proportion of the patients in the TIE-group were immunosuppressed on chronic steroid therapy, which has been identified as a risk factor for TIE [20]. The rate of permanent pacemaker implantation following TAVR is known to be high. These devices may be at risk of infection, which, when undiagnosed, could also lead to valvular endocarditis [1]. Several patients may go undiagnosed or be deemed unfit for surgery, without having their case discussed by an endocarditis team or even being referred to an experienced center. However, these results should encourage physicians to consider surgery in patients suffering from TIE and promote vigilance in patients following TAVR.

### 4.3. Limitations

This is a retrospective single-center study with the inherent limitation of such an analysis. The small number of patients is associated with a low power of statistical analyses. Owing to the small sample size, a multivariable analysis could not be performed. Furthermore, our study is a descriptive retrospective registry of patients with IE after SAVR and TAVR considered operable by the endocarditis team, and, therefore, it does not reflect the status of treatment of IE after TAVR. Patients who remained undiagnosed or were treated conservatively are out of the scope of this study. Further studies with longer follow-up are required.

## 5. Conclusions

To date, the current literature advises surgeons to err on the side of caution in cases of TIE; our results, however, indicate that patients suffering from SAVR–PVE and TIE present with comparable risk profiles and can be surgically treated with comparable results. The high rates of postoperative complications may be attributed to the disease and its severity. Endocarditis-specific risk scores should be included more frequently in the decision-making process, as they may predict the operative risk with more precision as compared to the classical scores. Surgery as a curative option should not be blatantly rejected, even in this high-risk cohort. However, the endocarditis-team approach and a patient-centered approach should always be considered.

## Figures and Tables

**Table 1:** **Baseline parameters.** BMI, body mass index; COPD, chronic obstructive pulmonary disease; LCOS, low cardiac output syndrome; LVEF, left ventricular ejection fraction; PVE, prosthetic valve endocarditis.

	SAVR–PVE (*n* = 98)	TIE (*n* = 22)	*p*-Value
Age, (years)	69 (58–76)	77 (70–80)	0.010
BMI (kg/m^2^)	25.7 (24.3–27.2)	25.7 (24.3–27.2)	0.645
Male (%)	88 (89.8)	18 (81.8)	0.285
NYHA class			0.073
NYHA I/II	21 (21.4)	1 (4.5)	
NYHA III/IV	77 (78.6)	21 (95.5)	
Charlson Comorbidity Index	5 (4–7)	7 (6–8)	0.005
Arterial hypertension (%)	76 (77.6)	19 (86.4)	0.560
Hyperlipoproteinemia (%)	50 (51.0)	15 (68.1)	0.235
Coronary artery disease (%)			0.966
One-vessel disease (%)	10 (10.2)	1 (4.5)	
Two-vessel disease (%)	15 (15.3)	2 (9.1)	
Three-vessel disease (%)	15 (15.3)	5 (22.7)	
PCI within 90 days (%)	6 (6.1)	1 (4.5)	1.000
Diabetes mellitus (%)	27 (27.6)	7 (31.8)	0.794
Chronic kidney disease (%)	26 (26.5)	10 (45.5)	0.120
Dialysis (%)	3 (3.1)	3 (13.6)	0.074
Creatinine clearance (mL/min)	54 (41–75)	49 (33–69)	0.201
Smoker (%)	22 (22.4)	7 (31.8)	0.410
COPD (%)	11 (11.2)	4 (18.2)	0.473
Pacemaker (%)	12 (12.2)	9 (40.9)	0.003
Atrial fibrillation (%)	23 (23.5)	7 (31.8)	0.423
Peripheral vascular disease (%)	9 (9.2)	5 (22.7)	0.133
Preoperative cerebral emboli (%)	14 (14.3)	3 (13.6)	1.000
Intravenous drug use (%)	2 (2.0)	0 (0.0)	1.000
HIV infection (%)	1 (1.0)	0 (0.0)	1.000
Chronic steroid therapy (%)	1 (1.0)	3 (13.6)	0.019
Previous malignancy (%)	13 (13.3)	5 (22.7)	0.320
Alcohol abuse (%)	8 (8.2)	2 (9.1)	1.000
Preoperative ventilation (%)	6 (6.1)	0 (0.0)	0.591
Preoperative LCOS (%)	9 (9.2)	1 (4.5)	0.687
Previous open cardiac surgery (%)	98 (100.0)	9 (40.9)	<0.001
Previous endocarditis (%)	11 (11.2)	1 (4.5)	0.693
Time to PVE (years)	3.7 (0.8–9.5)	1.2 (0.4–2.8)	0.001
Early PVE (%)	27 (27.6)	11 (50.0)	0.073

**Table 2:** Endocarditis-specific and -non-specific risk scores.

	SAVR–PVE (*n* = 98)	TIE (*n* = 22)	*p*-Value
**Non-Specific Scores**			
STS PROM	1.8 (1.6–2.1)	1.9 (1.4–2.2)	0.622
EuroSCORE II	52.1 (40.6–62.0)	45.4 (32.6–58.1)	0.207
**Specific Risk Scores**			
Risk E score	24.2 (19.5–38.2)	31.0 (21.3–39.6)	0.419
Endoscore	7.2 (4.5–12.6)	7.2 (4.3–12.6)	0.601
DeFeo Score	17 (14–21)	20 (16–23)	0.132
**Mid-Term Mortality Risk Score**			
ICE Score	37.4 (25.7–45.0)	39.8 (33.7–46.3)	0.207

**Table 3:** **Preoperative echocardiographic data.** LVEF, left ventricular ejection fraction.

	SAVR–PVE (*n* = 98)	TIE (*n* = 22)	*p*-Value
LVEF			0.554
≥50%	41 (41.8)	9 (40.9)	
31–49%	50 (51.0)	9 (40.9)	
≤30%	7 (7.1)	4 (18.2)	
Paravalvular leakage (%)	12 (12.2)	8 (36.4)	0.013
Aortic Stenosis			0.462
Mild to moderate (%)	2 (2.0)	0 (0.0)	
Moderate to severe (%)	10 (10.2)	4 (18.2)	
Aortic Regurgitation			0.072
Mild to moderate (%)	37 (37.8)	2 (9.1)	
Moderate to severe (%)	13 (13.3)	12 (54.5)	
Mitral regurgitation			0.085
Mild to moderate (%)	28 (28.6)	4 (18.2)	
Moderate to severe (%)	25 (25.5)	10 (45.5)	
Tricuspid regurgitation			0.346
Mild to moderate (%)	33 (33.7)	6 (27.3)	
Moderate to severe (%)	3 (3.1)	4 (18.2)	
Pulmonary hypertension (%)	12 (12.2)	5 (22.7)	0.306
Presence of vegetations (%)	76 (77.6)	13 (59.1)	0.104
Size of vegetations			0.022
<5 mm	13 (13.3)	3 (13.6)	
5–8 mm	21 (21.4)	6 (27.3)	
>8 mm	42 (42.9)	4 (18.2)	
Abscess (%)	39 (39.8)	6 (27.3)	0.335

**Table 4:** **Spectrum of pathogens.** BCNIE, blood-culture-negative infective endocarditis; CoNS, coagulase-negative staphylococci; MSSA, methicillin-susceptible *Staphylococcus aureus*; MRSA, methicillin-resistant *Staphylococcus aureus*. ^a^ Includes one polymicrobial infection: *Enterococcus faecalis* plus *Candida albicans*. ^b^ *Staphylococcus epidermidis* (SAVR–PVE, 4; TIE, 6), *Staphylococcus sciuri* (SAVR–PVE, 0; TIE, 1), and *Staphylococcus capitis* PVE, 1; TIE, 0). ^c^ Includes one polymicrobial infection: *Staphylococcus hominis* and *Staphylococcus epidermidis*. ^d^ *Streptococcus agalacticae* (PVE, 1; TIE, 0) and *Streptococcus anginosus* (SAVR–PVE, 1; TIE, 0). ^e^ *Streptococcus bovis* (SAVR–PVE, 1; TIE, 0). ^f^ Orally occurring Viridans group streptococci. *S. mitis* group, *S. oralis* (SAVR–PVE, 1; TIE, 0); *S. mitis* (SAVR–PVE, 2; TIE, 1); *S. salivarius* group, *S. salivarius* (SAVR–PVE, 0; TIE, 1); and *S. sanguinis* group, *S. sanguinis* (SAVR–PVE, 4; TIE, 0). ^g^ Includes two polymicrobial infection: *S. sanguinis* plus *S. aureus* and *S. oralis* plus *S. epidermidis*. ^h^ HACEK group, *H. parainfluenzae* (SAVR–PVE, 1; TIE, 0); *C. hominis* (SAVR–PVE, 0; TIE, 1).

Pathogen	SAVR–PVE (*n* = 98)	TIE (*n* = 22)	*p*-Value
**BCNIE**	27 (27.8)	1 (4.5)	0.024
**Gram-positive organisms**			
*Enterococcus* sp.	15 (15.3)	6 (27.3)	0.215
*Enterococcus faecalis*	13 (13.3) ^a^	5 (22.7)	
*Enterococcus faecium*	2 (2.0)	1 (4.5)	
Staphylococcus aureus	18 (18.4)	4 (18.2)	1.000
MRSA	1 (1.0)	0 (0.0)	
MSSA	17 (17.3)	4 (18.2)	
*Staphylococcus lugdunensis*	1 (1.0)	0 (0.0)	1.000
Other CoNS ^b^	5 (5.1)	7 (31.8) ^c^	0.001
*Streptococcus* sp.	12 (12.2)	2 (9.1)	1.000
*Streptococcus Group B* ^d^	2 (2.0)	0 (0.0)	
*Streptococcus Group D* ^e^	1 (1.0)	0 (0.0)	
*Orally occurring viridans streptococci* ^f^	7 (7.1) ^g^	2 (9.1)	
*Viridans streptococci not defined*	2 (2.0)	0 (0.0)	
*Micrococcus luteus*	1 (1.0)	0 (0.0)	1.000
*Lactococcus garvieae*	1 (1.0)	0 (0.0)	1.000
*Lactobacillus paracasei*	1 (1.0)	0 (0.0)	1.000
*Cutibacterium acnes*	11 (11.2)	1 (4.5)	0.693
*Parvimonas micra*	1 (1.0)	0 (0.0)	1.000
**Gram-negative organisms**			
*HACEK group* ^h^	1 (1.0)	1 (4.5)	0.334
*Moraxella catarrhalis*	1 (1.0)	0 (0.0)	1.000

**Table 5:** **Postoperative complications and outcomes.** ECLS, extracorporeal life support; IABP, intra-aortic balloon pump; ICU, intensive care unit; LCOS, low cardiac output syndrome; PMV, postoperative mechanical ventilation.

	PVE (*n* = 98)	TIE (*n* = 22)	*p*-Value
**Details of surgery**			
Cardiopulmonary bypass time (min)	203 (149–271)	127 (87–232)	0.005
Cross-clamp time (min)	134 (106–169)	95 (58–168)	0.003
Bentall procedures (%)	34 (34.7)	1 (4.5)	0.004
Repair of aortomitral curtain (%)	2 (2.0)	1 (4.5)	0.458
Aortic root enlargement (%)	0 (0.0)	1 (4.5)	0.183
Patch repair (%)	19 (19.4)	3 (13.6)	0.762
Aortic valve replacement			0.669
Biological prosthesis (%)	91 (92.9)	20 (90.9)	
Mechanical prosthesis (%)	7 (7.1)	2 (9.1)	
Concomitant procedures			
Mitral valve replacement (%)	18 (18.4)	5 (22.7)	0.499
Mitral valve repair (%)	2 (2.0)	0 (0.0)	1.000
Tricuspid valve replacement (%)	3 (3.1)	0 (0.0)	1.000
Tricuspid valve repair (%)	2 (2.0)	1 (4.5)	0.458
CABG procedure	8 (8.2)	1 (4.5)	1.000
**Morbidities**			
Adverse cerebrovascular events (%)	26 (26.5)	4 (18.2)	0.431
Severe bleeding with re-exploration (%)	20 (20.4)	4 (18.2)	1.000
Surgical site infection (%)	3 (3.1)	0 (0.0)	1.000
Tracheostomy (%)	10 (10.2)	3 (13.6)	0.709
Pacemaker implantation (%)	26 (26.5)	5 (22.7)	0.792
Renal replacement therapy (%)	18 (18.4)	8 (36.4)	0.091
LCOS (%)	16 (16.3)	7 (31.8)	0.137
Septic shock (%)	24 (24.5)	7 (31.8)	0.430
ECLS support (%)	17 (17.3)	2 (9.1)	0.521
IABP (%)	4 (4.1)	1 (4.5)	1.000
**Outcomes**			
In-hospital mortality (%)	26 (26.5)	2 (9.1)	0.098
Length of hospital stay (days)	19 (14–33)	23 (16–37)	0.234
Length of ICU stay (days)	5 (2–9)	4 (3–14)	0.953
Length of PMV (hours)	19 (12–75)	20 (13–113)	0.612

## Data Availability

The data presented in this study are available on request from the corresponding author. The data are not publicly available in accordance to national data safety guidelines.

## Abbreviations

BCNIE	Blood culture negative infective endocarditis
BMI	Body mass index
CABG	Coronary artery bypass grafting
CoNS	Coagulase-negative staphylococci
COPD	Chronic obstructive pulmonary disease
ECLS	Extracorporeal life support
EuroSCORE II	European System for Cardiac Operative Risk Evaluation II
HACEK	Haemophilus species, Aggregatibacter species, Cardiobacterium hominis, Eikenella corrodens, and Kingella species
HIV	Human immunodeficiency virus
IABP	Intra-aortic balloon pump
ICU	Intensive care unit
IE	Infective endocarditis
LCOS	Low cardiac output syndrome
LVEF	Left ventricle ejection fraction
MRSA	Methicillin-resistant *Staphylococcus aureus*
MSSA	Methicillin-susceptible *Staphylococcus aureus*
NVE	Native valve endocarditis
NYHA	New York Heart Association
PMV	Postoperative mechanical ventilation
PVE	Prosthetic valve endocarditis
SAVR–PVE	Prosthetic valve endocarditis following surgical aortic valve replacement
STS-PROM	Society of thoracic surgeons predicted risk of mortality
TAVR	Transcatheter aortic valve replacement
TIE	Prosthetic valve endocarditis following transcatheter aortic valve replacement
